# Viscoelastic cell model of sorting in the *dictyostelium discoideum* slug

**DOI:** 10.1371/journal.pone.0325141

**Published:** 2025-05-28

**Authors:** Erin Flowerday, Emily J. Evans, Christopher Grant, John C. Dallon

**Affiliations:** Mathematics Department, Brigham Young University, Provo, Utah, United States of America; Texas A&M University College Station: Texas A&M University, UNITED STATES OF AMERICA

## Abstract

Cell sorting and differential motion are key processes in the life cycle of Dictyostelium discoideum (Dd) and many other organisms. Here we develop a mathematical model and investigate the processes with computer simulations. The slug stage of Dd is modeled with ellipsoidal cells of two types which have viscoelastic properties. Using the force-based model we find that when the two cell types have different strengths of motive forces and or different degrees of directionality one cell type sorts to the front of the slug. These findings are consistent with previously published results using a different model formation. When one cell type is more directed than the other it will consistently sort to the front of the slug. Likewise, but less efficiently, when one cell type exerts greater motive forces than the other it will sort to the front of the slug. The most efficient and robust cell sorting due to differential motion is when both methods are employed.

## Introduction

Dictyostelium discoideum (Dd) is an interesting biological organism that can exhibit both a unicellular and multicellular form. They are amoebae that feed on soil bacteria or leaf matter bacteria, dividing freely in the presence of food. When starved, cell division stops, and cells transition from a unicellular to a multicellular state, triggered by the chemoattractant cyclic adenosine monophosphate (cAMP).

When cAMP is secreted, the cells move together to form mounds. Within these mounds, the cells differentiate into at least two cell types: prestalk (PST) and prespore (PSP) cells. Cell sorting occurs as the cell types position themselves in specific areas within the mound [1]. Due to each cell type’s expression of cell adhesion molecules, motility properties, and signaling pathways, polarization occurs within the mound. Prestalk cells are located towards the mound’s anterior region, whereas prespore cells are found in the posterior region [2].

Several possible mechanisms could cause the front-to-back pattern formation in the slug. These include: differential adhesion, cell differentiation, and differential motion. It is known that prestalk cells and prespore cells can change into the other cell type and are influenced by several chemical signals [3,4]. Yet, explaining the front-to-back pattern of cell differentiation is not enough. A mechanism for cell sorting is necessary [5], and because our model focuses on cell sorting, we do not consider cell differentiation in our model. There is evidence that prestalk cells chemotact more effectively towards cAMP than do prespore cells [6,7], and prespore cells adhere to one another more strongly than prestalk cells [8]; thus, both differential motion and adhesion could play a role. There are several theoretical models of cell sorting due to differential cell adhesion [9–11] but they predict that cell sorting should initiate with small clusters and eventually the more adhesive cells will be surrounded by the less adhesive cells which is not the case in Dd. Additionally, manipulation of adhesion molecules in Dd suggests that the role of adhesion in cell sorting is secondary [12]. Thus the focus of this paper is differential cell motion leading to cell sorting.

The mounds transform into a multicellular organism called a slug, which will migrate towards or away from stimuli. Moreover, cells within the slug migrate in a coordinated manner. Prestalk cells guide the slug’s movement, exhibiting chemotactic behavior towards environmental cues. This directional movement aids the slug’s navigation towards more favorable conditions for spore formation [1].

Furthermore, the cells culminate after migration, transforming into a fruiting body. The fruiting body consists of viable, environmentally resistant spore cells atop a long stalk composed of vacuolated dead stalk cells [2]. The fruiting body can be compared to a spore-producing organ of a fungus. Under favorable conditions, the fruiting body releases the spores, which grow into unicellular organisms, restarting the life cycle of the Dd cell [13].

Despite the extensive studies and modeling of Dd, how the slug moves and maintains the distribution of cell types in the slug is not fully understood. Here we aim to understand the mechanisms behind cell sorting and the front-to-back patterning within a Dd slug in a 3D environment by exploring cell motion and the interaction of cells. Although cell differentiation also plays a role in the sorting, it is not modeled in this work.

## Previous modeling

Pattern formation has been the subject of theoretical and mathematical models for many decades [14,15]. There are several good review articles that present a more in-depth study on modeling in pattern formation [16–20] and cell sorting [21–24]. Dd has also been the subject of extensive mathematical modeling to investigate the interplay between the chemical signaling and the cell motion [25–36]. In early aggregation Dd becomes chemotatically sensitive to cAMP as well as gaining the ability to relay the cAMP signal. Thus it forms an excitable medium that is reorganized with each wave of cAMP. In this manner, the Dd cell population can aggregate into centers and form slugs. The focus of this manuscript is the slug phase of Dd.

There have been several mathematical models of the slug stage of Dd [37–46]; of these [37,38,41,45,46] focus on cell sorting and pattern formation. The modeling reported here is most similar to [44,46] but also has similar aspects to [37,45].

The early model of Pate and Othmer [37] is a one-dimensional partial differential equation model which assumes three cell types, and three chemicals: a chemotactic agent, an agent causing differentiation, and an agent suppressing chemotaxis. They were able to reproduce many features of the front-back patterning in the slug. In [38], Umeda uses a one-dimensional continuum model as well which minimizes the free energy considering random motion, cell adhesion, and chemotaxis. Like us, Umeda does not consider cell differentiation in the model. Both of these previous models, while giving valuable information, are one-dimensional continuum models that do not focus on forces applied to individual cells. Our model is 3-dimensional, has discrete cells, and focuses on forces applied to individual cells.

In this work, we modify the model of Dallon and Othmer [44], who used incompressible ellipsoids with viscoelastic properties to investigate how the slug transmitted forces from individual cells to the substrate. Using a similar model formulation we investigate how cells sort in the slug due to differential motion.

The model of Maree *et al*., [41] is two dimensional, and uses a discrete continuum framework with each cell being composed of several automata. As in the previous models, they do not consider forces applied to individual cells. In their model they minimize the free energy of the system to determine the configuration of the cells.

The works [37,45,46] investigate to some degree cell sorting within the slug as do we. Pineda *et al*. [45] developed a differential equation model for the differentiation of two cell types. With this model they observed the proper pattern formation (i.e., distribution of cell types) in a mound and in a slug. Yet again, it is a continuum model which does not focus on forces applied by individual cells as we do. The model of Song *et al*. [46] considers sorting due to differential motion using a model which considers cell-cell adhesion sites surrounding a cell and the forces they exert. Thus of all these, only models [42–44,46] and the work here consider the forces of individual cells and only [46] and our work focus on cell sorting.

Our main goal, like [46], is to determine feasible strategies cells can employ to exhibit differential cell motion. In [46], they found that either different directionality in applying force or asymmetric force application resulted in differential cell motion with a combination of both giving a more robust strategy for differential cell motion. Our main objective is to test the conclusion of their work to see if we observe similar behavior with a viscoelastic model. This would indicate that the conclusions are robust and not model dependent. Several of these models reinforce the fact that chemotaxis is necessary, yet having several models that incorporate chemotaxis in different manners gives evidence that chemotaxis is the key ingredient and not the particular formulation in the model. Likewise, here we want to give evidence that differential direction and differentially applied forces are not an artifact of the model formulation in [46], but rather are robust ideas that lead to differential cell motion and they are not dependent on the model formulation or the manner they are implemented in the model. By providing a viscoelastic model that does not focus on the dynamics of cell-cell adhesion which gives similar results, we further support those strategies as being robust ones for the cell to employ.

## Model

In this study, a three dimensional model of the Dd slug will be developed to enhance understanding of the factors that initiate cell sorting within the slug. Since we are primarily interested in the differential motion within the slug, we do not consider the overall slug motion. There are no cell substrate motive forces, although all the cells are responding to a chemoattractant to the right. In other work [44], how the overall cell aggregate (the slug) gains tractional forces has been investigated. There it was shown that only the cells in contact with the substrate contribute to the overall motion of the slug. Thus, although cell substrate motive forces will alter the results they should be secondary effects.

### The cell

**Fig 1:**
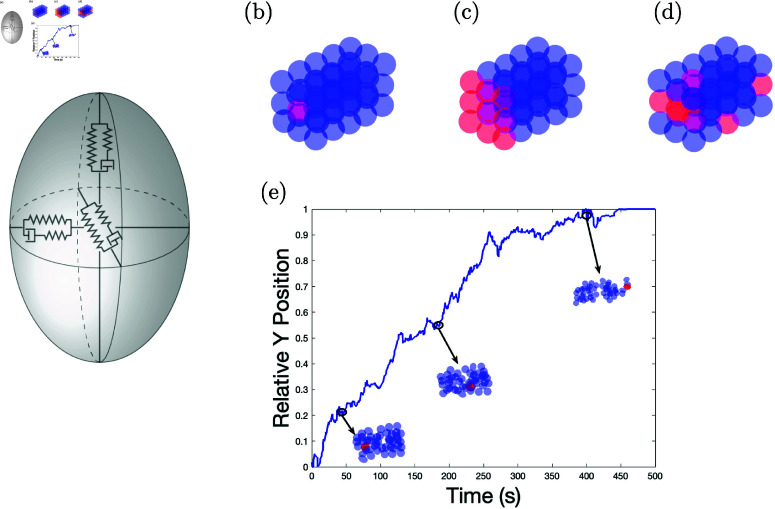
This figure depicts the model cell, initial configurations, and the relative position. Panel (a) shows a diagram representing the mechanical structure of the cell - an ellipsoid with three axes, where the length of each axis is governed by a standard linear solid. The three types of initial conditions are shown in (b), (c) and (d). In (b) there is one prespore cell in the posterior end of the slug, in (c) there are 9, and in d) they are randomly placed in the slug. Panel (e) show a graph of the relative position of the prestalk cells with three insets of the slug configuration at the time indicated. In panel (e) there is one prestalk cell with initial conditions as shown in (b), the prestalk cell force is 0.4 with ψ=45, and for the prespore cells the force is 0.01 with ψ=90.

The Dd slug is constructed from *n* individual cells. In the work presented here, as in the Dallon-Othmer model, each cell is assumed to be an oriented ellipsoid with constant volume. Therefore, an individual cell has three axes and the radii can vary in time. Each axis of the cell is a standard linear solid, consisting of two springs and a dashpot [47] (See Fig 1a).

The presence of the standard linear solid on each axis gives the cell viscoelastic properties. Since cellular volume is maintained, when stress is applied to one axis, the cell reacts by deforming in the other axes to maintain its volume.

Let *a*, *b*, and *c* be the lengths of the axes of the ellipsoid, *u*_*i*_ be the deformation of each axis, *f*_*i*_ be the force on each axis, *k*_*i*_ be the spring constant for the spring in parallel to the spring - dashpot, ${\bar{k}}_{i}$ be the spring constant for the spring in series with the dashpot, and $\mu_{i}$ be the dampening coefficient of the dashpot. The variable λ couples the equations and can be thought of as the pressure due to the cell membrane. Then the deformation is calculated using the equation,

$$u_{i}^{\prime }=\frac{\left(\frac{{\bar{k}}_{i}}{\mu_{i}}\cdot (f_{i}+\lambda -k_{i}\cdot u_{i})+\frac{df_{i}}{dt}\right)}{\left(k_{i}+{\bar{k}}_{i}\right)}$$ (1)

for i∈{a,b,c} where

$$\frac{dV}{dt}=\frac{d}{dt}\left(\frac{4\pi }{3}\frac{a}{2}\frac{b}{2}\frac{c}{2}\right)=0$$ (2)

and *V* is the volume of the ellipsoid.

### The slug

The forces within the system play a crucial role in generating cell movement, leading to sorting within the Dd slug. To simulate this phenomenon, we start with the cells organized in a three dimensional rectangular lattice (with some random perturbations) and specify which cells are prestalk and which are prespore. Since we are interested in sorting we have three configurations (as shown in Fig 1b–1d); two of which have the prestalk cells in the back of the rectangular array.

**Fig 2:**
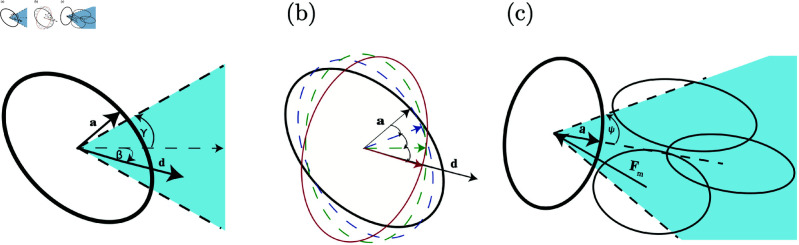
A 2D representation of cell rotation and motive force. Panel (a) depicts how the cell direction **d** is chosen in a γ cone about the *y*-axis. Panel (b) shows how the cell incrementally rotates to the new direction, aligning **a** with **d**. Finally, panel (c) shows how the motive force, $F_{m}$, is chosen. Each cell is polarized with its **a** axis being its direction of polarization. A cone of angle ψ is chosen, shown by the dotted lines, about the **a** axis, and the nearest neighboring cell center within the cone is where the motive force is applied. The cone shown in (a) gives the magnitude of random fluctuations in the chemoattractant signal, whereas, the cone in panel (c) represents how effectively the cell can track a direction.

We assume each cell is polarized with the direction of motion coinciding to the axis of the ellipsoid whose semi-axis is given by **a** (the length of the diameter is *a*). The model requires a direction stimulus **d** representing the direction of the chemoattractant stimulus. We assume the chemotaxis is directing the cells in the positive *y* direction. Thus **d** is a unit vector chosen in a specified cone angle, γ, around the *y*-axis (see Fig 2a). If γ is small there is less random fluctuation in the direction of chemoattractant signal.

If the orientation of the cell, the vector **a**, does not coincide with the direction stimulus given by vector **d**, the cell needs to rotate (or if the vectors are anti-parallel change front to back) until they do coincide (see Fig 2b). The cell rotates in specified angle increments until the two vectors align.

Since the Reynold’s number is low the acceleration term can be dropped and the position of the cells is governed by the following force equation:


$$M(𝐩){𝐩}^{\prime }=𝐅(𝐩)\;=F_{m}+F_{m}^{*}+F_{a}+F_{Bx}+F_{Bz}+F_{o}+F_{o}^{*}+F_{n},$$

where *M* represents the matrix which accounts for the drag on each cell. The vector **p** represents the position of all the cells given by


$$𝐩=[\begin{array}{c} p_{1}\\ p_{2}\\ \vdots \\ p_{n} \end{array}].$$

The vector 𝐅(𝐩) is the sum of all the forces acting on each cell; these include forces generated by the cell to move, forces acting on each cell by the environment, and forces from neighboring cells. In order to move, the cell generates a motive force, $F_{m}$, by pulling on a neighboring cell center. The cell is moved by the equal and opposite force denoted $F_{m}^{*}$ which provides the traction force for the cell. The substrate and slime sheath are modeled with imposed boundaries. We refer to these forces as environmental forces and denote them: $F_{Bx}$ and $F_{Bz}$. The adhesion forces, $F_{a}$, are due to cell adhesion. Finally, the rheological forces are due to the viscoelastic properties of each cell pushing neighboring cells away and are denoted $F_{o}$ for the forces pushing due to cell overlap, $F_{o}^{*}$ for the equal and opposite forces, and $F_{n}$ for the rheological force due to the cell nucleus.

The motive force is the force the cell applies to a neighboring cell to move in the direction **d**. We choose an angle ψ and define a cone about **d**. (There are two cone angles: one about the *y*-axis to give an upper limit on the magnitude of the perturbation about the *y*-axis defining **d** and one about **d** restricting the neighboring cells to which the motive force is applied.) The motive force is applied to the nearest cell within the cone angle. It is calculated based on the direction from a cell to its nearest neighbor within the cone angle’s range (see Fig 2c). Its force component is given by


$$F_{m}=\kappa \cdot {\hat{𝐯}}_{\text{ij}}$$

where κ is the motive coefficient, which specifies the magnitude of the motive force. The vector $v_{ij}$ is the vector pointing from the center of cell *i* to the center of the nearest neighbor cell *j* falling within the cone angle ψ, and ${\hat{𝐯}}_{\text{ij}}$ is the unit vector in the same direction.

**Fig 3:**
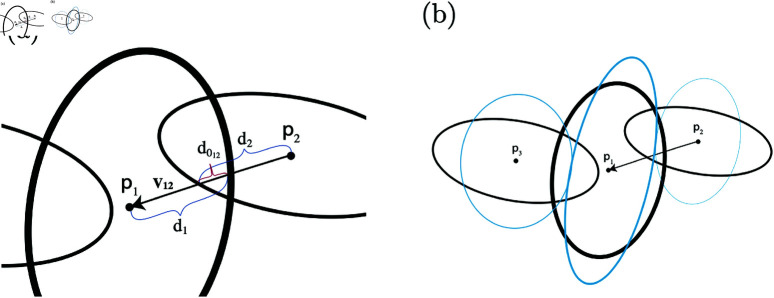
A 2D representation of the progression of three overlapping cells before and after deformation adjustments. (a) Shows three cells initially overlapping, focusing on the region of overlap between cell 1 and 2. (b) Illustrates the cells before (in black) and after deformation (in blue), where adjustments have been made to account for the overlap, demonstrating the new positions and shapes of the cells.

To determine the drag, adhesive, and rheological forces we first need to determine the cell overlaps. For each cell, overlaps are determined by comparing the distance between cell centers against the sum of the distances from the cell centers to the ellipsoid’s defining cell boundary, as seen in Fig 3, where *p*_1_, *p*_2_, and *p*_3_ represent the center positions of cells 1, 2, and 3, and $v_{12}$ is the vector between the centers of cell 1 and 2.

Let *d*_*i*_ be the distance between the center of cell *i* and its edge in the direction of $v_{ij}$ and similarly for *d*_*j*_. Let $d_{0_{ij}}$ be the overlap distance in the direction of $v_{ij}$ (see Fig 3). We define $d_{0_{ij}}$ as follows,


$$\left\{ \begin{array}{cc} d_{i}+d_{j}\leq \|v_{ij}\| & \text{no overlap occurs}\\ d_{0_{ij}}=d_{i}+d_{j}-\|v_{ij}\| & \text{otherwise}. \end{array}\right.$$

The calculation of the overlap distances is the first step in accounting for the eventual cell deformation and is important in determining the adhesion and rheological forces. If the cells overlap, to determine the rheological forces we calculate the cell shapes with less or no overlap but do not reshape the cell at this juncture, and only use the information to determine the impinging force. The goal is to find the shape for each cell so that the new cell boundary intersects with the point in the middle of the line measured by $d_{0_{ij}}$ for each overlapping cell while keeping the cell center and volume the same. There are three scenarios: overlap with a single neighboring cell, overlapping with two neighboring cells, and overlapping with three or more neighboring cells. There are three variables defining the length of each axis and the volume constraint. For each overlapping cell there is one constraining point. When there is one overlapping cell there are three variables and two constraints —the system is underdetermined. We add a constraint to minimize the sum of the axes. For two overlapping cells the system is exactly determined and we can solve the system. For three or more overlapping cells the system is overdetermined and we use a least squares solution.

More specifically points for the new boundary are determined by adjusting the length *d*_1_ to be $d_{1}^{new}$ in the following manner. In the case with a single neighboring cell


$$d_{1}^{new}=\left\{ \begin{array}{cc} ||v_{12}||-0.7d_{2} & \text{if}d_{0_{12}}\geq ||v_{12}||\\ d_{1}-\frac{d_{0_{12}}}{2} & \text{otherwise.} \end{array}\right.$$

The first equation is when the neighboring cell boundary has moved beyond the cell center. For the case with two neighboring cells, for each neighboring cell *j* we define


$$d_{1}^{new}=d_{1}-\frac{d_{0_{1j}}}{2}.$$

In the final case with three or more neighboring cells we use a non-linear least squares method to fit an ellipsoid that approximately goes through the points which are defined in the same manner as the two neighbor case.

For the one and two neighboring cell cases we use a nonlinear optimization algorithm and for the three or more neighboring cell case we use a nonlinear least-squares optimization [48]. To maintain the stability of the system, in the nonlinear least squares optimization, the radii are only allowed to deform a certain amount. The upper bound to which the cell radii can grow is 6μm and the lower bound is 4μm.

Cell adhesion is modeled with two components: a component tangential to the cell membrane modeled as a drag force and a component normal to the cell membrane. When cells interact with each other via cell-cell adhesions their membranes can slide past each other but are more resistant to separation due to the dynamics of the attaching and detaching of the adhesion sites. Both components of the force are modeled as functions of the contact area between the two cells. The contact area between the two cells in our scenario could be assumed to be the surface area of the ellipsoid which is protruding into another cell. This would not give the same area for both overlapping cells. Thus for simplicity in determining this contact area we assume the cells are spheres with radii *d*_*i*_ and *d*_*j*_ and determine the area of the circle of intersection between the two spheres, which is given by


$$\zeta =\pi \frac{2(d_{i}+d_{0_{ij}}+d_{j}{)}^{2}d_{i}^{2}+2(d_{i}+d_{0_{ij}}+d_{j}{)}^{2}d_{j}^{2}+2d_{i}^{2}d_{j}^{2}-(d_{i}+d_{0_{ij}}+d_{j}{)}^{4}-d_{i}^{4}-d_{j}^{4}}{4(d_{i}+d_{0_{ij}}+d_{j}{)}^{2}}$$

if they intersect and 0 if they do not. The adhesive component normal to the cell membrane is given by


$$F_{a}=\left\{ \begin{array}{cc} \alpha \cdot \zeta \cdot {\hat{𝐯}}_{\text{ij}} & \text{if}0<d_{0_{ij}}\leq 0.1(d_{i}+d_{j})\\ \alpha {\left(\frac{0.3(d_{i}+d_{j})-d_{0_{ij}}}{0.2(d_{i}+d_{j})}\right)}^{2}\bar{\zeta }\cdot {\hat{𝐯}}_{\text{ij}} & \text{if}0.1(d_{i}+d_{j})<d_{0_{ij}}<0.3(d_{i}+d_{j})\\ 0 & \text{otherwise} \end{array}\right.$$

where α is the scaling factor, which quantifies the strength of adhesion between two cells. We restrict the area of overlap to not exceed $\bar{\zeta }$ and then quadratically ramp down the magnitude of the adhesive force when the overlap is too large. Thus $\bar{\zeta }$ is the area of the circle when $d_{0_{ij}}=(\mathrm{.1}(d_{i}$  +  *d*_*j*_)). Given that cells grab onto each other when in close proximity, we calculate the adhesion forces with enlarged cells. That is we use the values 1.4*d*_1_ and 1.4*d*_2_ for the radii. Thus adhesion forces are present even when the cells are not touching but are close to each other.

We assume the slug is confined to a square box of infinite length in the *y* direction. Thus there is an upper and lower boundary in the *x* and *z* directions. The magnitude of the boundary forces is proportional to the distance from the cell center to the boundary. Let the cell center be at $𝐩=(p_{x},p_{y},p_{z})$ and r=5μm be the cell radius; then the magnitude of the boundary force in the *x* direction is


$$F_{Bx}=\left\{ \begin{array}{cc} r\rho & \text{if}p_{x}<0\\ \rho (r-p_{x}) & \text{if}0<p_{x}<r\\ \rho (35-r-p_{x}) & \text{if}35-r<p_{x}<35\\ -r\rho & \text{if}p_{x}>35\\ 0 & \text{otherwise} \end{array}\right.$$

and similarly for the *z* direction.

The rheological force component prevents cells from moving through each other. If one cell, cell 1, moves into another cell, cell 2, the rheological forces repel the cells. They are a combination of the nucleus force and the overlap forces. The first overlap force applied to cell 1 is calculated based on the calculated deformation of cell 2 (as explained previously). The second overlap force, denoted with an ‘*’, is the equal but opposite force which is applied to cell 1 in response to the deformation of cell 1 and the force it applies to cell 2. The nucleus force repels the cells away from each other if the cell nuclei impinge on each other. Thus when two or more cells overlap the overlap forces are applied and when the overlap is great enough for the nuclei to impinge the nucleus forces are added to the overlap forces. Since the nucleus is a very rigid structure the nucleus forces are exponential. The equation is given by


$$F_{rheological}=F_{nucleus}+F_{overlap}+F_{overlap}^{*}.$$

The nucleus repelling force is symmetric and so there is no need for an equal and opposite force.

The nuclei in Dd are indeed small (we assume a diameter of 4 microns) and more malleable than many other eukaryotic cells. Our model formulation requires some additional force of the nuclear type. To prevent cells from passing through each other the cells need to stiffen as the cell centers get close. Yet in order to allow them to deform at realistic forces the viscoelastic properties of the cells as currently modeled are not sufficient to prevent cells from passing through each other. The problem is real in that cells resist being punctured by other cells, yet our model formulation is not as robust as the biological system in this aspect. This highlights the need to test the basic mechanisms with different model formulations for validity.

After the cells’ adjusted radii are determined, the forces that cause the cells to deform are calculated. The force required for the cells’ deformation is calculated as the overlap force,


$$F_{overlap}=[\begin{array}{c} f_{a}\\ f_{b}\\ f_{c} \end{array}]=[\begin{array}{c} -(k_{a}+{\bar{k}}_{a})(a^{new}-a)\\ -(k_{b}+{\bar{k}}_{b})(b^{new}-b)\\ -(k_{c}+{\bar{k}}_{c})(c^{new}-c) \end{array}]$$

where $f_{i}\text{for}i\in \{a,b,c\}$ is the force applied in the direction of the {a,b,c} axis respectively. The spring constant $k_{i}\text{for}i\in \{a,b,c\}$ is for the spring found in the linear solid model on each axis, connected in parallel; whereas, ${\bar{k}}_{i}\text{for}i\in \{a,b,c\}$ is the spring constant found in the linear solid model on each axis, for the spring connected in series with the dashpot. The parameters ${\text{a}}^{\text{new}}$, ${\text{b}}^{\text{new}}$, and ${\text{c}}^{\text{new}}$, are the diameter of the ellipsoid as calculated above to avoid overlap and *a*, *b*, and *c* are the diameters of the ellipsoids.

The forces are derived from the relaxation function of the viscoelastic cell axes which are modeled as Standard Linear solids [47]. Recall that the relaxation equation is the force of the element when it is instantaneously deformed. If the deformation takes place at time *t* = 0, $F_{overlap}$ simplifies into the sum of the spring constants multiplied by the deformation.

When cells come into close proximity, the nucleus force is also included,


$$F_{nucleus}=\left\{ \begin{array}{cc} 10(e^{3(\frac{1}{\left\|v_{ij}\right\|}-\frac{1}{4})}-1) & \text{if}\|{𝐯}_{\text{ij}}\|<4\\ 0 & \text{otherwise} \end{array}\right.$$

where 4 represents the diameter of the nucleus of a Dd cell [49].

The final component in the equation is the mass matrix, *M*. It is a block matrix composed of *M*_*ii*_ and *M*_*ij*_ blocks. It accounts for the fluid drag and the adhesion drag that the cell experiences. It is a 3*n* by 3*n* matrix where *n* is the number of cells, given as follows: For *i* = *j*,


$$M_{ii}=\left(\chi_{ii}\mu_{f}+\sum_{k\neq i}\chi_{ik}\mu_{cell}^{ik}\right)[\begin{array}{ccc} 1 & 0 & 0\\ 0 & 1 & 0\\ 0 & 0 & 1 \end{array}].$$

For i≠j,


$$M_{ij}=-\chi_{ij}\cdot \mu_{cell}^{ij}[\begin{array}{ccc} 1 & 0 & 0\\ 0 & 1 & 0\\ 0 & 0 & 1 \end{array}],$$

where $\chi_{ij}$ is the normalized contact area of the cell with its neighboring cell, which is


$$\chi_{ij}=\frac{\zeta }{4(5{)}^{2}\pi }$$

and $\chi_{ii}$ is the normalized contact area the cell has with the fluid and is calculated as,


$$\chi_{ii}=\max\left\{0,1-\sum_{j\neq i}\chi_{ij}\right\}.$$

Here $\mu_{f}$ is the fluid drag coefficient, $\mu_{cell}^{ij}$ is the cell-cell drag contribution for each pair of cells, and $4(5{)}^{2}\pi$ represents the surface area of the spherical cell.

The position of the cells is determined by solving the force equations utilizing the ARKODE solver package in C++ [50]. Changes to the cell shape (changing the radii of the ellipsoids) are handled numerically by lagging the cell shape and then updating it once the new cell locations are determined. The cell shapes are updated by determining the overlap as previously described and then evolving the equations for the coupled standard linear solids along each of the principal axes of the ellipsoid as described in Eqs 1 and 2 assuming no force is applied to the elements. Thus the cell shapes are determined by the overlap equations and then they are allowed to relax towards their spherical steady state shape until they arrive at the current time.

Let *t*>*t*_0_, **r** be the radii of all the cells (half axes of the visco-elastic elements), **f** be the forces in the direction of the radii (the viscoelastic elements), and let


$$M(𝐩,𝐫){𝐩}^{\prime }=F(𝐩,𝐫)$$

be the equation describing the evolution of the cell positions. Furthermore, let


$$G(𝐩,𝐫,{𝐫}^{\prime },𝐟(𝐩,𝐫))=0$$

be the equation describing the evolution of the cell radii. We numerically solve the equation starting from *t*_0_ in the following manner:


$$M(𝐩(t),𝐫(t_{0})){𝐩}^{\prime }(t)=F(𝐩(t),𝐫(t_{0}))$$

$$G(𝐩(t),𝐫(t),{𝐫}^{\prime }(t),0)=0.$$

## Results

For this study, simulations were run to understand cell sorting and differential cell motion within the slug, especially the front-to-back pattern observed between prestalk and prespore cells. The prestalk cells were colored red, and prespore cells were colored blue. For all simulations shown here, a slug consisting of *n* = 45 cells, with one prestalk cell, thus *n*_*st*_ = 1, is configured as seen in Fig 1b. The prestalk cell was initially positioned on the posterior end of the slug to observe its movement to the slug’s anterior region. Several simulations were run with the other two initial conditions (Fig 1c and 1d) verifying the general results hold in those cases as well. We graph the relative *y* position of the prestalk cells. Let *y*_1_ be the leftmost *y* coordinate, *y*_2_ be the rightmost *y* coordinate of the slug, and *y*_0_ be the average *y* coordinate of the prestalk cells. Then the relative *y* position is defined to be $\left(y_{0}-y_{1})/(y_{2}-y_{1}\right)$ (see Fig 1e). Unless otherwise noted, the set of parameters used in the simulations is given in Table 1.

**Table 1:** The default parameters used in this study unless otherwise noted (* can be *a*, *b*, or *c*).

Default Parameters					
General Parameters		Prespore Parameters		Prestalk Parameters	
Parameter	Value	Parameter	Value	Parameter	Value
*n*	45	$k_{*}$	$100{\text{g/s}}^{2}$	$k_{*}$	$100{\text{g/s}}^{2}$
*n*_*st*_	1 or 9	${\bar{k}}_{*}$	$100{\text{g/s}}^{2}$	${\bar{k}}_{*}$	$100{\text{g/s}}^{2}$
*dt*	0.001 s	$\mu_{*}$	160 g/s	$\mu_{*}$	160 g/s
$\mu_{f}$	0.1 g/s	$\mu_{cell}$	20$\mu_{f}$	$\mu_{cell}$	20$\mu_{f}$
ρ	$10{\text{g/s}}^{2}$	κ	$0.01\text{g}\mu m^{2}$/s	κ	$0.4\text{g}\mu m/{\text{s}}^{2}$
α	$0.1\text{g}\mu m/{\text{s}}^{2}$	ψ	90	ψ	30

Short term passive viscoelastic properties of cells have been measured [51–55]. Our simulation considers the shape changes of the cell to be due to active remodeling of the cytoskeleton during cell migration and would give very different results from the typical measurements on passive cells with small displacements. Thus we choose parameters for the viscoelastic properties of the cell which are similar to those in [44]. There a typical cell surrounded by ten other cells would deform 2.5 microns in four minutes and relax to its original shape in about seven minutes.

Slug sizes are typically several orders of magnitude larger than the ones we simulate here. Due to computational limitations, the stochastic nature of the simulations and our desire to run several realizations for each case, and ease of visualization we have run our simulations with a small number of cells per slug. We have run some simulations on slugs with 250 cells which give results consistent with those reported here. We see no reason that scaling the simulations up will change the results or conclusions.

**Fig 4:**
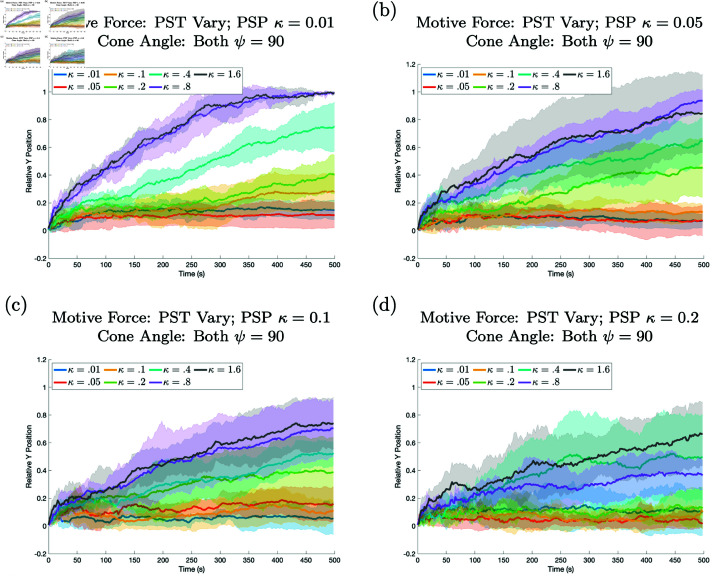
Results when the prestalk cell motive force is varied are shown. In all panels there is some sorting, but there is less sorting as the difference between the motive forces of the two cell types decreases. The relative position of the prestalk cell for different motive forces is plotted against time. In each panel the motive force, κ, for the prestalk cell is varied while different panels show simulations where the prespore cells’ motive force is changed. In (a) prespore cell motive force is fixed at κ=0.01, in (b) κ=0.05, in (c) κ=.1, and in (d) κ=0.2. In all cases the cone angle is 90 for both cell types. All other parameters for both cell types are the same. For all figures unless otherwise specified, the middle line is the mean of 5 realizations and the shaded region shows the standard deviation and the initial configuration is one prestalk cell.

A simulation was run to establish a base case in which there was no differentiation between the prespore and prestalk cells apart from their coloring. Both types of cells were assigned the same parameters, and their sorting was observed. Each cell was given a cone angle of ψ=90 degrees and an active force of κ=0.01. The results in Fig 4a (the blue line) show that no sorting was achieved within the slug when the prestalk cell had the same motive force as the prespore cells (κ=0.01). The same is true for κ=0.05, 0.1, and 0.2 (see Fig 4).

### Varying one parameter

Further investigations were carried out to determine the specific impact of solely adjusting one parameter. Thus in these simulations the only difference between prestalk and prespore cells is different values of one parameter. We found that varying only the motive force or the directionality can result in cell sorting, and varying only the directionality resulted in slow sorting.

**Active force.** First we varied the active force of prestalk cells, κ. In Fig 4 the results of the prestalk cell with active force of κ=0.01,0.05,0.1,0.2,0.4,0.8 and 1.6, while prespore cells maintained an active force of κ=0.01 in a), κ=0.05 in b), κ=0.1 in c), and κ=0.2 in d) are shown.

Notably, all other cell parameters are the same for both cell types throughout the simulations. The outcomes revealed that when the active force of the prestalk cell is sufficiently large when compared to the active force of the prespore cells the prestalk cell will sort (see Fig 4 and S1 Movie). It seems that there is some threshold value as well as a functional dependency on the force of the prespore cells for sorting to occur. As the motive force of the prespore cells increases the sorting of the the prestalk cell for a fixed motive force decreases. Thus greater cellular forces can cause sorting but it is dependent on the force of the surrounding cells.

These results are consistent with Song *et al*. [46], who found that altering cell strength did not yield differential motion, but altering the cell forces in an asymmetric manner could yield differential motion. The motive force in the model here is inherently asymmetrical since it only pulls on one cell.

**Fig 5:**
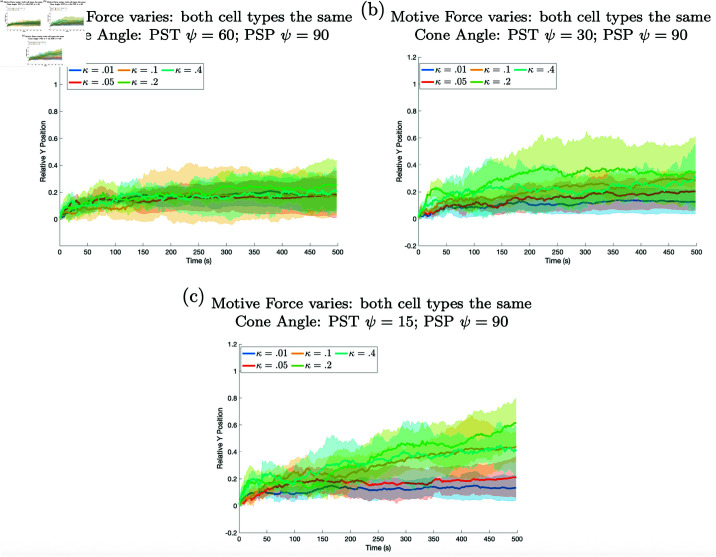
Results when the two cell types have different cone angles but the same motive force. Decreasing the cone angle promotes sorting for higher motive forces, but in all cases there is not much sorting. In panel (a) the cone angle for the prestalk cell is 60, in (b) 30, and in (c) 15. The cone angle for prespore cells is fixed at 90 degrees for all simulations in this figure. The motive force for the prestalk cell and prespore cells is the same but varies from curve to curve.

1 **Directionality.** To investigate the effects of the cone angle on cell sorting within the slug, the magnitude of the prestalk cell’s cone angle was reduced. It is expected that a smaller cone angle restricts the cell’s outreach area, leading to more directed movement. Experiments were conducted with cone angles of ψ=15,30, and 60 degrees for the prestalk cell while prespore cells maintained a cone angle of ψ=90 degrees and the active force of both cells remained the same but was varied. Fig 5c shows that altering the cone angle enough, thus giving the cell more directed motion, causes the cell to start sorting to the front when the active force is great enough (see S2 Movie). Changing the cone angle alone yields sorting, albeit more slowly than when the active force is changed. Again, this is in agreement with the results in Song *et al*. [46] where they found that by reducing the cone angle the cells exhibited differential motion.

### Changing combinations of parameters

In these simulations more than one parameters is different for the two cell types. Thus we investigate how the combination of different mechanisms influences differential cell motion. We find that different directionality in combination with different motive forces is the most effective mechanism for cell sorting.

**Fig 6:**
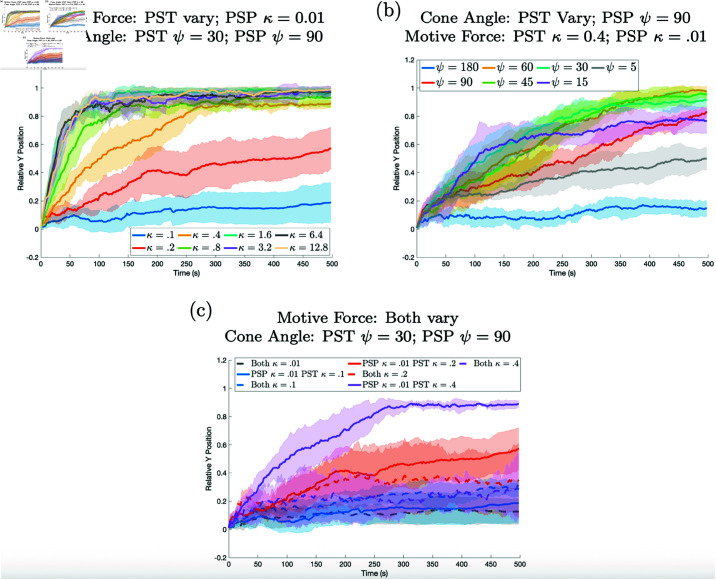
Results when the cone angle and motive force strengths are varied. More directed cells with greater motive force sort more quickly. In panel (a) the cone angles are fixed at ψ=30 for prestalk cells and ψ=90 for prespore cells. The motive force κ is varied for prestalk cells and fixed at κ=0.01 for prespore cells. In panel (b) the cone angle for prestalk cells is varied and the motive force is fixed at κ=0.4. For prespore cells ψ=90 and κ=0.01. In panel (c) the curves for Fig 5b (dashed lines) are compared with the curves for panel (a) (solid lines). In this case, when there is no difference in the motive forces for the two cell types (dashed lines), cell sorting does not occur.

**Fig 7:**
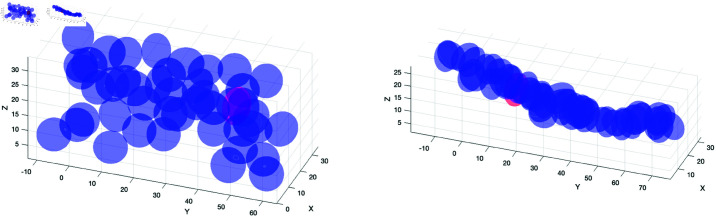
The positions of the cells within the slug in one realization for two different simulations (simulations shown in Fig 6c). On the left the prestalk cell has an active force of 0.4 and prespore cells have an active force of 0.01. On the right both cell types have an active force of 0.4. In both simulations the prestalk cell and prespore cells have cone angles of 30 degrees and 90 degrees respectively. The panels show the cell positions at time 120 seconds.

**Combination of directionality and motive force.** In these simulations we first fix the cone angle at different values for each cell type and vary the motive force. Then we fix the motive force at different values and vary the cone angle. In Fig 6a the results are shown for the varying motive forces and in panel b) the results are shown for varying cone angle. As can be seen in panel a) and S3 and S4 Movies, increasing the motive force causes faster sorting. Panel b) shows there is an optimal cone angle for the best sorting. When the cone angle is too small the sorting is less efficient. This is likely due to the fact that with a smaller cone angle there are fewer possible cells to pull on. Panel c) indicates that it is not only the prestalk motive force that is important, but the prespore motive force also plays a role. Or rather, the magnitude of the difference is important with larger differences in motive forces resulting in faster sorting. Finally, when the majority of the cells have larger motive forces the slug becomes very elongated as can be seen in Fig 7, S2 and S3 Movies.

**Fig 8:**
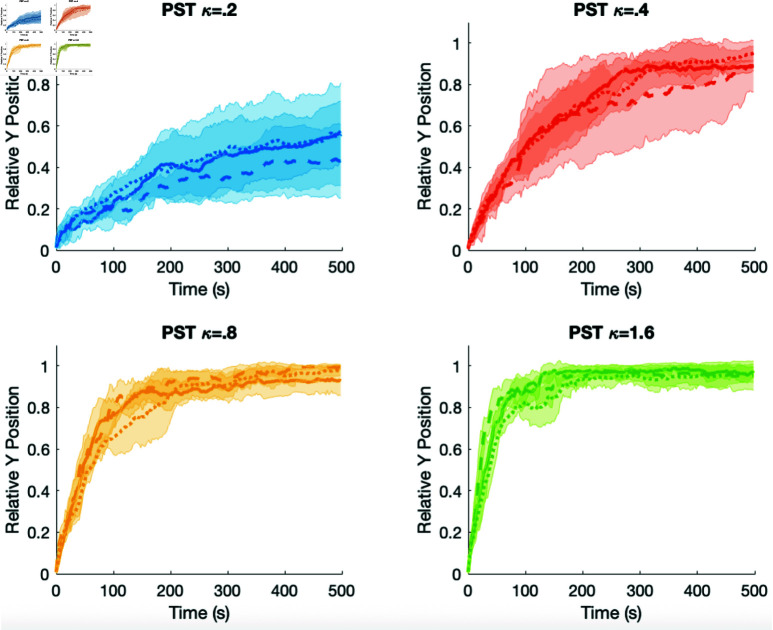
Comparison of results when the prespore cells are stiffer (dashed lines), the same (solid lines), and less stiff (dotted lines) than the prestalk cell. For the prestalk cell, κ varies and ψ=30, and for the prespore cells κ=0.01 and ψ=90. The relative position of the prestalk cell’s *y* coordinate is the *y* axis and time is the *x* axis in seconds.

**Directionality, force, and body stiffness.** We also ran simulations where the viscoelastic properties of the cells are different. We incorrectly thought that softer prespore cells would allow faster sorting. The more rigid cell would be able to move more readily through the softer cells. It turns out that different viscoelastic properties of the two cell types do not appreciably affect the sorting (see Fig 8). In these simulations the prespore cells have ψ=90, motive force κ=0.01 and are stiffer or softer than the prestalk cell which has ψ=30 and various values for the motive force. In Fig 8 the solid lines show results from standard simulations where the viscoelastic properties of both cell types are the same. The dashed lines show simulations where the prespore cell properties are twice that of the prestalk cell (stiffer) and the dotted lines show simulations where prespore cells have parameters which are one half that of the prestalk cells (softer). That is, the spring constants in the standard linear solid, $k_{*}$ and ${\bar{k}}_{*}$ and the coefficient for the dash pot, $\mu_{*}$ for the prespore cells are 2 times that of the prestalk cell values for all axes (* being *a*, *b*, or *c*) for the dashed case and are 0.5 times those of the prestalk cells for the dotted case. As usual, when the prestalk cell motive force is too low (0.2) there is slower sorting. As the motive force for the prestalk cell increases the less stiff prespore cells (dotted lines) slightly delay the sorting. For the stiffer prespore cells (dashed lines) when the force is low the sorting is delayed more than the softer prespore cells, but as the motive force increase there is no difference. When the prespore cells are stiff the slug quickly becomes less dense which helps promote the sorting. This is likely due to the ratio of the adhesion forces and the rheological forces. The stiffer cells push more strongly as the cells impinge on each other but the adhesion forces are never changed. Thus the greater rheological forces when compared to the same adhesive forces cause the slug to become less cohesive.

**Fig 9:**
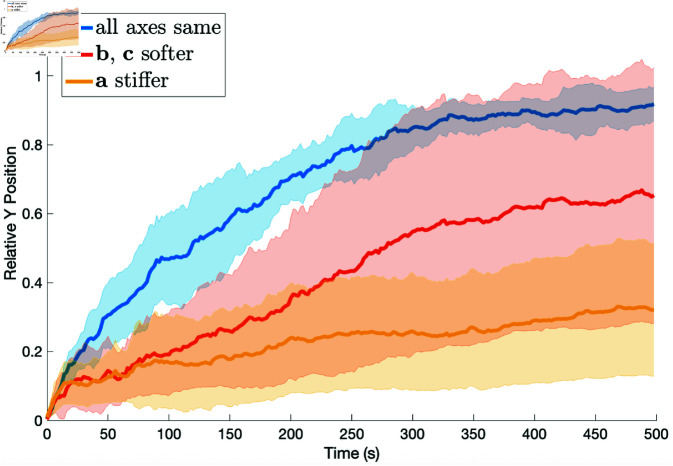
Comparison of results when the viscoelastic properties of prestalk cell axes vary, the same (blue), b and c are softer (red), and a is stiffer (gold). The parameters are ψ=30 and κ=0.4 for the prestalk cell and κ=0.01 and ψ=90 for the prespore cells. The softer axes have $k_{*}$, ${\bar{k}}_{*}$ and $\mu_{*}$ one half of normal and the stiffer case has $k_{*}$, ${\bar{k}}_{*}$ and $\mu_{*}$ two times normal. The relative position of the prestalk cell’s *y* coordinate is the *y* axis and time is the *x* axis in seconds.

Next we tried simulations where the axes of the prestalk cell have different viscoelastic properties. The axis aligned in the direction of motion **a** was either stiffer or the other two axes were more compliant. Fig 9 shows that having all axes the same gives the best results and stiffening the **a** axis is least effective. It was interesting that in this set of simulations, the other two initial conditions (Fig 1 c and d) were less sensitive to the variation in the viscoelastic properties than the initial condition with one prestalk cell. Yet the final relative position of the prestalk cells with the other initial conditions remained bounded by the relative position of initial condition with one prestalk cell. That is, when all axes had the same properties the one prestalk cell sorted better than any other case and when the **a** axis was stiffer, the one prestalk case had the smallest relative position compared with all the other simulations.

**Fig 10:**
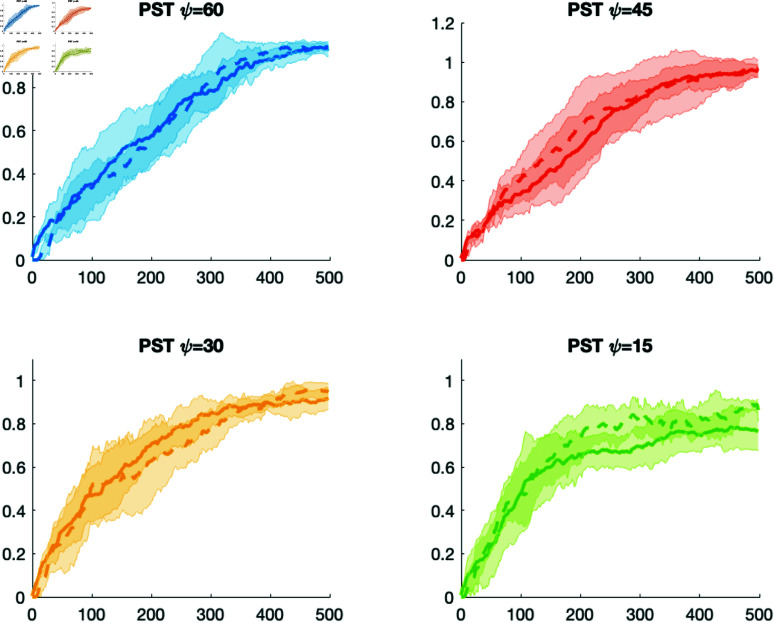
Comparison of results when the prestalk cell is spherical (solid lines) and slightly elongated (dashed lines). Prestalk cell’s ψ varies and κ=0.4. Prespore cells’ κ=0.01 and ψ=90. The relative position of the prestalk cell’s *y* coordinate is the *y* axis and time is the *x* axis in seconds. Slightly elongated means the radius of the *a* axis of the ellipsoid is 1.25 times as long as the spherical cell and the radii of the other axes are divided by $\sqrt{1.25}$ to maintain the same volume.

**Body shape, directionality, and motive force.** We also investigated the case where the prestalk cell has a resting state which is elongated, theorizing that it would more easily move through the prespore cells whose resting state was spherical. Thus we ran a set of simulations where the prestalk cell has a resting state where the radius of the *a* axis of the ellipsoid is 1.25 times as long as the spherical cell and the radii of the other axes are divided by $\sqrt{1.25}$ to maintain the same volume, resulting in a slightly elongated prestalk cell. In addition the cone angle of the prestalk cell was varied and the motive force was κ=0.4. The cone angle of the prespore cells was 90 and the motive force was κ=0.01. The results are shown in Fig 10. The solid line is when both cells are spherical at resting state and the dashed line is when the prestalk cell is slightly elongated. As can be seen in the case where the prestalk cell cone angles are 60, 45, and 30 there is little difference in the sorting. When the cone angle is 15 the elongated prestalk cell may sort slightly faster.

## Discussion

The most effective method found for cell sorting was a combination of more directed cells which are stronger. By differentiating both parameters, ψ the size of the cone angle for the motive force and κ the magnitude of the motive force, the prestalk cells showed differential motion in the midst of the prespore cells thus moving to the anterior end of the slug. As previously mentioned, prestalk cells chemotact in response to cAMP more effectively than prespore cells [6,7]. Additionally, there is evidence that prestalk cells move faster [56] and (under certain circumstances) polarize more readily [57] when stimulated with cAMP than do prespore cells. The results of these biological experiments are consistent with the mechanisms proposed in our model. Our results agree with the results of Song *et al*. [46] where they found that the most efficient method for directed motion was a combination of stronger asymmetrical forces and more directed motion (a smaller cone angle). Finding the same results in two disparate force-based models of cell motion suggests that the conclusion is robust and not model dependent. Furthermore, either condition individually can result in preferential cell motion and cell sorting but the combination of both strategies gives the most efficient and robust results. The motive force of one cell type must both exceed a threshold and the difference between it and the motive force of the other cell type must be large enough for cell sorting to occur. This is reasonable since the motive force must be large enough for cell motion and larger than the other cell type for it to move ahead. When both cell types have large motive forces, the high opposing forces make the numerical scheme harder to solve.

A more streamlined cell shape did not affect sorting and non-isotropic viscoelastic properties inhibited cell sorting. These results could be due to the model restrictions that the cells are always ellipsoidal in shape. Relaxing this condition would be more realistic and may give different results for these two scenarios.

Finally, of the three types of initial conditions simulated, the one with one prestalk cell results in the most effective cell sorting. The relative position measure was more dramatic because the center of mass for one cell is its center which can be at the front edge of the slug. The center of mass for many cells is naturally farther back and is not at the very front edge of the slug. Additionally, in the initial condition where all the prestalk cells started in the back, as a block of cells, the sorting would start out more slowly. The prestalk cells would act like a "bulldozer" and push all the front cells until they gradually separated. Once they separated the individual cells sorted forward more easily.

Differential cell motion and cell sorting are important phenomena which need more study to be fully understood. Mathematical modeling is a useful tool which can give valuable insight into how these processes work.

## Supporting information

S1 Appendix Additional parameter details. (PDF)

S1 MovieSlug with no differential motion.This movie shows one realization when the cone angles of the two cell types are the same, θ=90, and the motive force for both cells is the same, κ=0.2 (see Fig 4d).(MP4)

S2 MovieSlug with some differential motion due to more directed PST cells.This movie shows one realization when the cone angles of the two cell types differ, θ=90 for PSP cells and θ=15 for PST cells. The motive force for both cells is the same, κ=0.2 (see Fig 5c).(MP4)

S3 MovieSlug with differential motion due to both more directed and stronger PST cells.This movie shows one realization when both the cone angles and the strength of the two cell types differ. The cone angles are θ=90 for PSP cells and θ=30 for PST cells. The motive force for PSP cells is κ=0.01 and for PST cells κ=0.8 (see Fig 6a).(MP4)

S4 Movie Larger slug with differential motion due to both more directed and stronger PST cells.This movie shows one realization for a slug with 250 cells when both the cone angles and the strength of the two cell types differ. The cone angles are θ=90 for PSP cells and θ=15 for PST cells. The motive force for PSP cells is κ=0.01 and for PST cells κ=0.4.(MP4)
