# Supplementary material for: Viscoelastic cell model of sorting in the dictyostelium discoideum slug
Source: PLoS One. 2025 May 28;20(5):e0325141. doi: 10.1371/journal.pone.0325141 (PMC12118998; doi:10.1371/journal.pone.0325141)
Supplement: S1 Appendix — (PDF) [file pone.0325141.s001.pdf]

S1 Appendix

We assumed the drag force for a sphere in water at low Reynolds number is $\mu_s = 3\pi 2r\mu v$ where $\mu = 1\text{cP}$ is the dynamic viscosity of water, v is the velocity, and $r = 5$ microns is the radius of a cell. We further assume that the cell drag is equal to 10 times μ_s, thus we set $\mu_f = .1$ in units of g/s. $\mu_{cell} = 20\mu_f$. The viscoelastic properties of the cell are chosen to be of the same order of magnitude as those in Dallon & Othmer [1].

References

1. Dallon J, Othmer H. How cellular movement determines the collective force generated by the Dictyostelium discoideum slug. Journal of Theoretical Biology. 2004;231:203–222.
